# DNA methylomic homogeneity and heterogeneity in muscles and testes throughout pig adulthood

**DOI:** 10.18632/aging.104143

**Published:** 2020-11-20

**Authors:** Min Zheng, Shijun Xiao, Tianfu Guo, Lin Rao, Longyun Li, Zhiyan Zhang, Lusheng Huang

**Affiliations:** 1 State Key Laboratory for Pig Genetic Improvement and Production Technology, Jiangxi Agricultural University, Nanchang, China

**Keywords:** pig, DNA methylation, WGBS, homogeneity, heterogeneity

## Abstract

DNA methylome pattern is significantly different among tissues, ages, breeds, and genders. We assessed 20 methylome and transcriptome data in *longissimus dorsi* (LD) or testicles from Bamaxiang (BMX) and Large White pigs (LW) by deep sequencing technology. We identified ~55.7M CpGs and 5.30M, 0.20M, 1.20M, and 0.16M differential CpGs (*P*<0.01) between tissues, ages, breeds, and genders, respectively. Interestingly, 7.54% of differentially methylated regions (DMRs) are co-localized with promoters, which potentially regulate gene expression. RNA-seq analysis revealed that 23.42% CpGs are significantly correlated with gene expression (mean |r|=0.58, *P*<0.01), most of which are enriched in tissue-specific functions. Specially, we also found that the methylation levels in promoters of 655 genes were strongly associated with their expression levels (mean |r|=0.66, *P*<0.01). In addition, differentially methylated CpGs (DMCpGs) between breeds in HOXC gene cluster imply important regulatory roles in myocytes hypertrophy and intermuscular fat (IMF) deposition. Dramatically, higher similarity of methylation pattern was observed within pedigree than across pedigrees, which indicates the existence of heritable methylation regions. In summary, a part of CpGs in promoter can change its methylation pattern and play a marked regulatory function in different physiological or natural environments.

## INTRODUCTION

DNA methylation plays a mechanistic role in embryonic development [1–3], aging [4–7], and diseases [1, 7, 8], these changes alter the availability of DNA to the binding proteins and the spatial organization of chromatin that would either enhance or repress gene transcription [9–13]. 5-methyl cytosine is enriched in CpG dinucleotides, and their methylation status can be copied from the parental strand to the offspring strand during cell replication, which attracts more attention than other classes of methylation pattern. Human genome assembly contains about 3 × 10^7^ CpG dinucleotides, and about 21.8% CpGs (5.6 out of 25.71 million) are dynamic among cell types, and 15.4% CpGs (4.1 out of 26.5 million) are strongly differentially methylated among tissues [14, 15]. It has been found that methylation level is highly associated with aging. And about 2% CpG sites show hypermethylation or hypomethylation with ageing [16, 17]. The overarching pattern of DNA methylation changes may activate or repress specific transcriptional programs [9].

Recent studies have reported breed-specific, tissue-specific, and age-specific methylated CpGs in pig genome [18–22]. Epigenetic atlas of various pig skeletal muscle and adipose tissues from different breeds have been investigated, and the methylation status within promoters is negatively correlated with mRNA and miRNA expression [19, 21, 23]. Long et al. identified 9234 DMRs in skeletal muscles between young and middle-aged pigs using methylated DNA immunoprecipitation sequencing (MeDIP-seq) data, and they detected a significant negative correlation (r=-0.21, *P*=3.19×10^-7^) between the methylation of gene and its expression [22]. Environmental changes are also associated with genome methylation levels. For example, Long et al. reported DNA-methylation-mediated gene expression alterations of Tibetan pigs in low-altitude acclimation [24]. These studies showed personalized and common characteristics of pig methylome, but there are challenges in clarification of the relationship between methylome and expression, and the effect of differential methylome on phenotypic variation under identical or discrepant growth stage, physiological and natural environment.

DNA methylation represses transcription by directly and indirectly inhibiting the binding process of transcription factors and promoters [1]. However, recent studies have also found that methylation can promote the transcriptional activation of gene [11], and a part of transcription factors (TFs) like to bind the methylated motif [10].

On the other hand, embryonic DNA methylation is reprogrammed in vertebrates, but a considerable fraction of mammalian genomes might potentially bypass the demethylation process during preimplantation and PGC reprogramming [25]. DNA methylation in the germ line of adults can be inherited intergenerationally [25, 26]. Twins study on methylation and their methylation correlations suggested that an average of up to 20% variance was explained by additive genetic factors across whole methylation sites [27]. But it is unclear which individually methylated/demethylated regions are passed on to or reappear in adult tissues of offspring.

We herein survey global genome DNA methylation level using high-depth WGBS from 1 to 9 year-old BMX pigs and 7-months LW pigs, with the aim to 1) draw a high-resolution dynamic methylation map based on different genders, breeds, tissues, and age status, 2) to explore the relationship between DNA methylation and tissue-specific function at transcriptomic and phenotypic level, 3) to detect the inheritable methylation regions whose methylated status transmit from the parent to the offspring generations. Pork is the main source of edible meat, and pigs have been widely used in human medicine as the large model mammal for organ transplantation and disease researches. The systematic investigation of methylomic homogeneity and heterogeneity under different factors will provide an important theoretical basis for pig breeding and human medical models.

## RESULTS

### The distribution of global CpGs and methylation level

**Figure 1:**
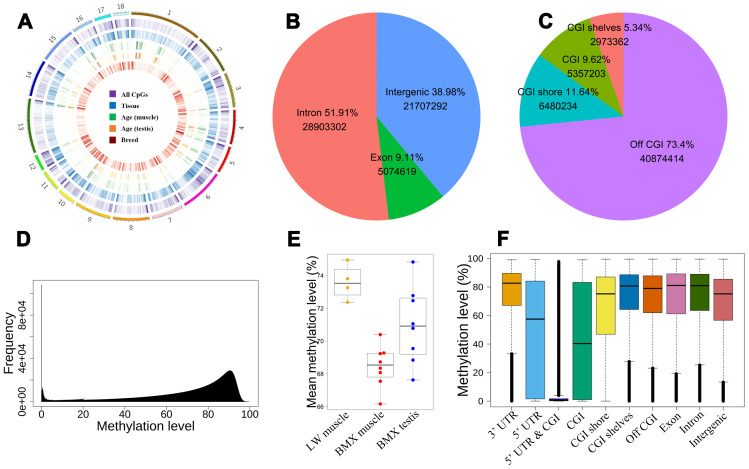
**The distribution of CpGs.** (**A**) The global distribution of CpGs and dynamic CpGs. From outside to inside, density plot show the global DMCpGs between tissues, dynamic CpGs with age (muscle and testis), and DMCpGs between breeds. (**B**) The intergenic and intragenic CpGs. (**C**) Positions of CpGs relative to CGI. (**D**) The distribution of CpG methylation levels. (**E**) Mean global methylation level of 20 samples. (**F**) The distribution of methylation levels in different genome regions.

In total, we acquired 2.24Tb WGBS data with an average of 46X genome coverages in 20 samples for BMX and LW, of which 55,685,213 autosomal CpGs (both strands of DNA) were detected. These CpG sites are unevenly distributed across the genome, with the preference of locating at the ends of chromosomes, and abundantly clustered in local locations (Figure 1A).

Integrating genome annotation information, we detected the proportion of these CpG sites distributed in introns, exons, and intergenic regions are 51.9%, 9.11%, and 38.98%, respectively (Figure 1B). Further analysis uncovered that 9.62% of 55,685,213 CpG sites are enriched in CpG islands (CGIs), of which 11.64% CpGs are located in CGI shores (Figure 1C).

We retained about 35M CpGs whose coverage ≥10 and ≤300 in half of samples to investigate the global methylation level. The global methylation levels formed a skew bimodal distribution with mean 70.5% (Figure 1D). In LD, the global methylation level was higher in LW than in BMX. Comparing results between tissues, the methylation level in testis was higher than in LD for BMX (Figure 1E). Based on the annotated genes and CGIs in pig genome, we also showed the distribution of methylation levels over ten regions (Figure 1F). Lower mean methylation levels (~50%) were found in 5’ UTRs and CGIs, and methylation levels in the two regions had a larger inter-quartile (~0.8). In addition, overlap of 5’ UTRs and CGIs (5’ UTR-CGI) had an extremely low methylation level (mean 7.65%) (Figure 1F and Supplementary Table 1). Focusing on hypomethylation and hypermethylation CpGs, we explored the distribution of 1,147,532 hypomethylated CpGs (methylation level was lower than 10%) and 5,743,199 hypermethylated CpGs (higher than 90%). More than half of hypomethylated CpGs are localized in CGIs or CGI shores, and the hypomethylated sites are also enriched in 5’ UTRs (Fisher’ exact test odds=2.80, *P*=2.2e-16). Our results revealed hypermethylated global genome and local hypomethylation in genome and further discovered highly variable methylation level and hypomethylation in 5’ UTRs and CGIs.

To further investigate relation between gene expression and methylation, we focused on the correlation between methylation level in the promoter regions and gene expression as most of CGIs are located in gene promoters in mammalian. We herein calculated the mean methylation level in 1000 bp promoter regions (800 bp upstream of TSS to 200 bp downstream of TSS). We retained 7558 genes’ promoters which have at least ten CpGs in our filtered data. About 6.8% (655 out of 7558) mean methylation level of promoters were strongly correlated with genes’ expression (Spearman correlation, *P*<0.01, mean |r|=0.66). The results suggested that gene promoter methylation plays an importantly regulatory role in gene expression in the adult tissues.

### Differentially methylated CpGs between tissues

To reveal tissues or organs specific DMCpGs relating to structural and functional elements in adulthood pigs, we detected the methylomic differences between LDs and testes from young to elder stages. The joint results of three differential analyses under BGLMM framework identified ~5.3 million DMCpGs (9.52% out of captured autosomal CpGs, *P*<0.01, Figure 1A) which belonged to 1,466,027 discrete DMRs following Matthew D.S et al. [15]. Focusing on the DMRs in promotors, we uncovered 110582 DMRs positing within 1^st^ exon or 1Kb upstream of genes and rigorously screened out 4978 DMRs (53627 DMCpGs) which included at least 5 synclastic DMCpGs. Correlation analysis showed 7361 DMCpGs and 5197 DMCpGs were negatively and positively correlated with genes’ expression, respectively (mean correlation coefficient was -0.59, *P*<0.05 and 0.57, *P*<0.05). The methylation status of DMRs in adult tissues may have positive and negative effects on the expression levels of 8% and 11% of genes (Supplementary Figure 3). DMRs of 1362 genes were detected to be significantly differentially expressed between two tissues (Wilcoxon signed rank test, *P*<0.01, Supplementary Table 2), among which DMRs locating in genes’ promoters displayed higher differential expression tendency (Fisher’s exact test, odds ratio=1.27, *P*=2.82E-12). There were no significant differences in expression of the remaining 3616 genes. These results implied that a part of DMRs continuously regulated the tissue-related function through all adulthood, and others might be of spatiotemporal characteristics or other unknown regulatory mechanisms.

For the known functional and significantly differentially expressed 873 genes, we performed Gene ontology (GO) analysis and found that 549 genes were enriched into 101 GO terms (*P*<0.05) in three categories including biological process (65 terms), cellular component (19 terms) and molecular function (17 terms). Muscle tissue-related function term includes muscle contraction, muscle filament sliding, myofibril assembly and skeletal muscle cell differentiation. Testis specific functional ontology includes meiotic DNA double-strand break formation, sister chromatid cohesion, spermatid development and acrosomal membrane (Supplementary Table 3). In addition, 70 genes were enriched into a biological process of positive regulation of transcription from RNA polymerase II promoter (GO:0006936). The results showed that DMCpGs were associated with tissue-specific cell differentiation and gene expression.

**Figure 2:**
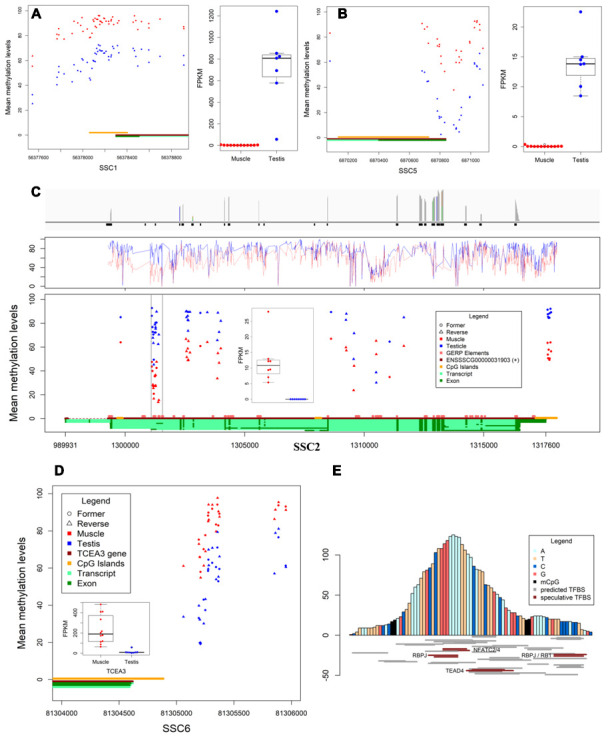
**Differentially methylated regions and expression.** (**A**, **B**) The DMR located in promotors and expression of *SPACA1* and *MEI1*, respectively. (**C**) The differential transcription levels and methylation levels of *TNNT3* in muscles and testes. Bar plot shows the reads coverage of RNA-seq data from muscles (top), line chart shows the methylation levels of *TNNT3* in both tissues (middle), the scatter plot shows the methylation levels and distribution of DMCpGs in *TNNT3*, and box plot shows the transcription levels of *TNNT3* in both tissues (bottom). (**D**) Methylation level in DMR of *TCEA3’s* upstream was positively correlated with expression. (**E**) Prediction of transcription factor binding sites for conserved sequences.

The mechanisms of DNA methylation in regulating gene expression are very complex and diverse. Herein we report three different DMRs in promotor whose methylation levels are negatively correlated with gene expression (Figure 2A–2C), including DMR co-localized with CGI (*SPACA1*), differential DNA methylation valley nearby the transcriptional start site (*MEI1*), and DMR co-localized with conservative sequence (*TNNT3*). Besides, we also detected a certain number of positive correlations between methylation in upstream proximal DMRs (*TCEA3*) and its expression (Figure 2D). It supports the current research results that methylated regulatory elements activate gene expression [10, 11].

Normally, gene expression is regulated by gene promotor methylation. Interestingly, DMR on chromosome 2 (*chr2:1301111-1301408*) correlated with expression was located in *TNNT3* rather than in its promotor region (Supplementary Figure 2). This region is probably not the transcription initiation site in muscles based on RNA-seq data (Figure 2C). Conservative analysis for 21 eutherian mammals revealed an 83bp constrained EPO-low-coverage element (*chr2:1301181-1301263*) was co-localized in the DMR, and the methylation levels of CpGs between constrained region and the ~100bp flanking region were significantly different in two tissues. Thus, the 83nt core sequence has been browsed to AnimalTFDB3.0 (http://bioinfo.life.hust.edu.cn/AnimalTFDB/#!/), and 64 pieces of information was retrieved (*P*<10^-4^). However only five expressing predicted transcription factor genes (including *NFATC2*, *NFATC4*, *RBPJ*, *RB1* and *TEAD4*) were identified in the two tissues (Figure 2E). The results showed the constrained element is an alternative promotor or tissue-specific enhancer in *TNNT3* of pig.

### Dynamic CpGs with age

**Figure 3:**
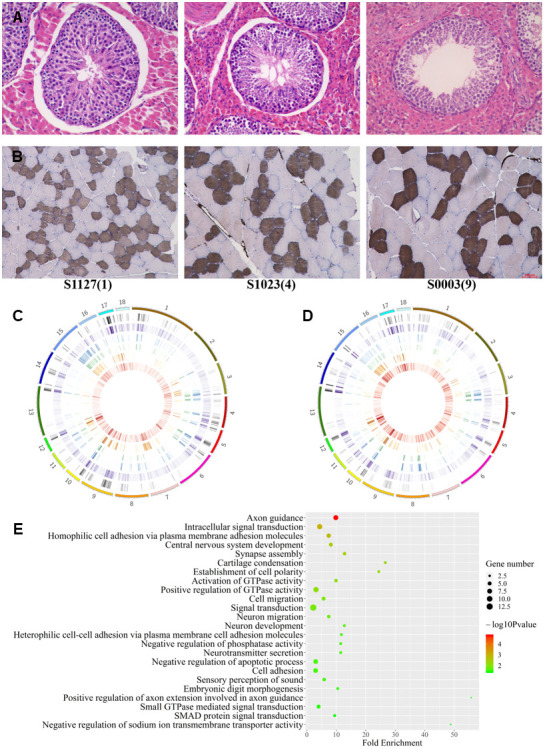
**Differences among age groups.** (**A**, **B**) Testis and muscle tissue sections with HE and ATPase staining, respectively. (**C**, **D**) The distribution of autosomal DMCpGs between any two age groups. From outside to inside, density plot show the results of six differential analyses under 9vs1Ymuscle, 9vs1Ytestis, 9vs4Ymuscle, 9vs4Ytestis, 4vs1Ymuscle, and 4vs1Ytestis, respectively. (**E**) GO terms for genes with highly dynamic CpGs which were shared in two tissue.

In order to clarify the physiological tendency in LD and testis under three age status, the sections of muscles and testes were stained with ATPase and HE, respectively (Figure 3A, 3B). Histological sections of LDs showed reducing of slow muscle fiber ratio and hypertrophic multinucleated muscle cells with age (Figure 3B). Histological sections of testes showed a decrease in the number of germ cells and Sertoli cells, and a shrinkage in seminiferous tubules with aging (Figure 3A). the 9Y testes showed typical histological aging characteristics [28].

We herein compared the methylation level among ages to see that if the distinct phenotype was regulated by methylation. Our methylomic data showed no significant difference in methylomic entropy and global methylation level across age groups (ANOVA, *P*>0.05), but weak decreasing tendency of methylation level was observed (methylome level in 1Y, 4Y, and 9Y age groups were 72.94%, 70.95%, 69.72%, respectively).

Focusing on some specific CpGs, we detected 9821 and 9759 dynamic CpGs with age using BGLMM in LDs and testes respectively. The dynamic CpGs are unevenly distributed on the genome, of which 766 (~7.8%) have been found in both tissues. The CpGs varied consistently in both tissues, which suggested methylation hotspot with ageing (Figure 1A). The dynamic CpGs showed more hypomethylation with age (57%) than hypermethylation (43%), and 69% of shared dynamic CpGs were hypomethylated with age. The Short Time-series Expression Miner (STEM) analysis revealed several differentially dynamic patterns in CpG methylation with age (Supplementary Figure 4).

Using methylation difference threshold algorithm (minimum methylation difference ≥0.3), we identified 147159 and 294439 dynamic CpGs varying with age in LD and testis, respectively (Figure 3C, 3D). We validated 56% dynamic CpGs identified by BGLMM algorithm. A total of 43,655 dynamic CpGs were shared in the two tissues and more hypermethylation with age than hypomethylation was showed. The lack of regular MDCpGs supports the previous hypothesis of stochastic epigenetic drift with aging. However, we captured a gene set including 93 genes which harbored at least 50 intragenic DMCpGs in both tissues. The shared genes enriched into 14 biology process terms which refer to neuron migration and development, signal transduction, and cell adhesion (Figure 3E). Among them, at least 16 homologous genes (including *AKT3*, *ARNT*, *CDC42BPA*, *CDH4*, *COL11A1*, *CTNNBL1*, *MYO1D*, *PRKCE*, *PSD3*, *PTPN4*, *RAD51B*, *RGS7*, *SLC24A3*, *TGFB2*, *TMEM117*, *UNC5D*) are listed in the aging gene database (http://genomics.senescence.info/genes/).

### Differentially methylated CpGs in LD between breeds

**Figure 4:**
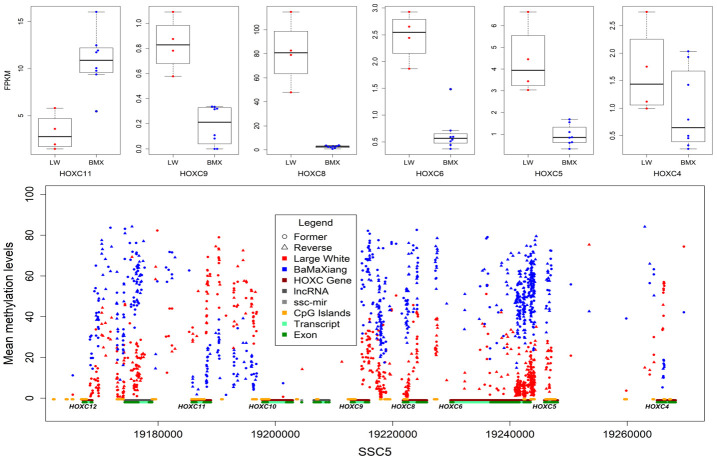
**DMCpGs in HOXC gene cluster and differentially expressed HOXC genes.**

Focusing initially on our muscle samples of two pig breeds, we captured 1147591 dynamic CpGs, of which 63.31% are distributed within 25959 genes. We detected 7876 co-directional methylated CpGs in 605 genes which were significantly differential expressed (Wilcoxon signed rank test, *P*<0.01) in LDs between two breeds. The *HOXC* gene cluster (*ssc12:24764000-2489000*) exhibited rich differential methylation sites and 38.77% of which were located in CGIs. Further analysis of RNA-seq data indicated 6 out of 9 *HOXC* family genes were differentially expressed (Figure 4), the expression levels of *HOXC4-6*, *HOXC8*, *HOXC9* in LD of LW were higher than that of BMX, and the *HOXC11* was in opposite in LD of two breeds (Figure 4). This results suggested that intragenic and intergenic DMRs may affect expression of the host gene. We focused on *HOXC8* which was highly expressed in LD of LW. Muscles may require HOXC8 protein for full activation of muscle-specific gene expression [29]. Up regulation of *HOXC8* in LD suggests its role in lineage development of muscle satellite cell (MSC) into trunk muscles [30].

Other two genes, *ZIC1* and *BMP5*, were highly expressed in LD of LW. BMP signaling regulates MSC-dependent postnatal muscle growth [31], and *ZIC1* is a marker of adipose tissue browning in humans, which is defined as the conversion of white fat into brown fat [32]. Compared with LW pigs, BMX pigs have lower eye muscle area (13.63 cm^2^ vs 51.47 cm^2^) and higher intramuscular fat content (IMF, 2.93% vs 1.89%) [33, 34]. The results suggested a set of highly specific-expressed genes with hypomethylation in LD of LW are beneficial for LD hypertrophy and maintenance and are not conducive to the deposition of IMF.

### DMRs between two pedigrees

**Figure 5:**
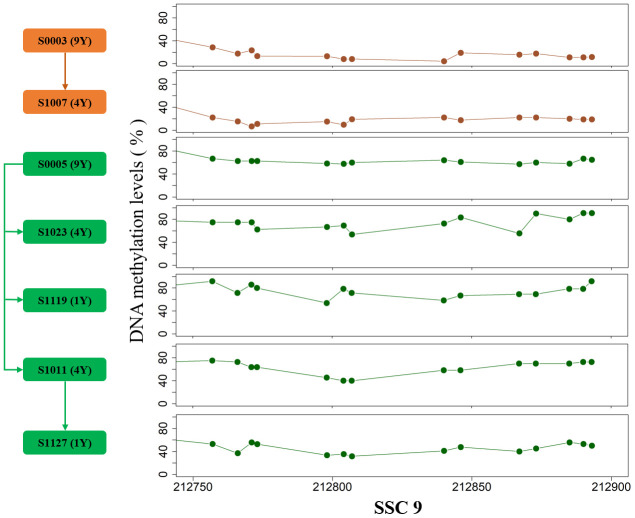
**The DMR on SSC9 between two pedigrees. The arrows indicate the parent-child relationship.**

The breed-specific methylomic pattern suggested that partial DNA methylation characteristics were stably transmitted within single breed. To verify intergenerational inheritance, we further compared the methylation levels between two pedigrees, and a total of 64 and 17 inheritable DMRs were identified in testes and muscles, respectively (Adjusted false positive <0.01, Methods, Supplementary Table 4). The parental methylation patterns in genome regions are transmitted to the offspring, strongly supporting the heritability of partial methylated or demethylated sites in adult tissues (Figure 5). We carefully analyzed an about 130bp DMR on SSC9 in testes which harbored 14 DMCpGs. After correcting the effect of sequencing depth on methylation level by the posterior probability of beta distribution [14], the methylation pattern differences between pedigrees are more than 0.3, suggesting intergenerational or transgenerational epigenetic inheritance.

### Dynamic CpGs between genders

We found that 155211 of CpG sites (567289 DMRs) in these experiments are strongly differentially methylated between genders (Minimum methylation difference ≥0.3), of which 69 are located in gene promotor DMR and 3419 are intragenic. Differential expression tests for DMR genes suggest no significant or weak differences (Wilcoxon signed rank test, *P*>0.05) in expression level whether they are in the promoter or intragenic regions.

## DISCUSSION

Meat quality and fecundity are both important economic traits in farm animals. Their heritabilities are relatively low, and they often differ significantly between breeds. To better understand the genetic mechanisms behind these traits, we conducted epigenetic studies on their related tissues including muscles and testes. In this study, the precious pig samples were acquired with broad age spanning from 1Y to 9Y under the same feeding condition. We systematically surveyed dynamic methylomes in a group of tissues, breeds, ages and genders throughout pig adulthood. Emphasis was placed on the DMRs that continued to affect gene expression and regulate specific function with age.

A large quantity of DMRs located in promoter region significantly negatively or positively correlated with gene expression. We speculated that a part of transcription factors bind to an hypomethylation environment and the others prefer to an hypermethylation environment. Several mechanisms of DNA methylation repressing transcription have been clarified [1]. Recent studies have reported a class of TFs that prefer to bind to some methylated sequences over the corresponding unmethylated sequence [10]. Homeodomain factors including HOCX11 and HOXB13 bind to the methylated recognition sequences, which lead to an increase in transcription [10]. SUVH proteins bind to methylated DNA and recruit the DNAJ proteins to enhance proximal gene expression [11]. The results illustrate the positive correlation between methylation and expression as TFs preferably bind to methylated motif.

CGIs occupy two thirds of all mammalian promoters, they are rarely methylated, which is a signature of active promoters [35, 36], and they display higher transcriptional activity than non-CGI promoters [37]. Our results showed that CGI in promoters may affect transcriptional activity through methylation or demethylation in themselves (*APACA1* and *TOPAZ1*) or flanking sequences (*MEI1*). However, CGIs located within gene bodies show the greatest number of DNA methylation differences between different somatic cells and tissues [38]. The CpG sites in introns of particular genes often play a role of their secondary enhancers [39]. The methylation levels of intergenic and intragenic DMRs in HOXC gene cluster co-located with CGI were negatively correlated with gene expression. The results indicated that the methylation of “orphan” CGIs are associated with gene’s expression. Interestingly, we found that 5' UTR-CGI regions had an extremely low methylation level. These hypomethylated CpGs were located in 923 genes, of which only 206 had expression levels (FPKM) of more than 1, and the other 717 genes were at low expression or silenced in all samples. The results suggest that demethylation of 5' UTR-CGI regions cannot independently activate or inhibit gene expression. We speculate that it may provide a flanking environment for the promoter, or that it may play an important role in high-dimensional interaction or long-distance regulation.

DNA sequence alone cannot explain hundreds of cell types in a complex multi-cellular organism. Cell-specific expression genes and epigenetic status provide a measure to estimate the number and proportion of cell types. Combining tissue-specific expression data with differentially methylated status in different samples, we could infer the number of opening DNA molecules in bulk genomes. We successfully found marker regions and corresponding genes (*APACA1* and *TNNT3*) by analysis of the methylation status of tissue-specific genes. Although the methylation levels are consistent with the phenotypes, higher-depth WGBS data and more samples are still required for accurately calculating the proportion of functional cells. Meanwhile, the two marker genes are associated with important human diseases. SPACA1-deficient male human and mice are infertile with abnormally shaped sperm heads reminiscent of globozoospermia [40, 41]. *TNNT3* is associated with nemaline myopathy and distal arthrogryposis [42]. Understanding the epigenetic regulatory mechanisms are beneficial for research or medical treatment of human diseases. Thus, dynamic DNA methylation level in cell-specific expressed genes reflects the changes of cell composition and function in tissues and helps reshape the trajectories of growth, development, aging, and disease. In addition, disease model pigs are widely studied for human diseases and are even considered as donors for human organ transplants. Understanding the epigenetic mechanism of pigs is bound to lay a biological foundation for the treatment of human diseases. At the same time, it can provide an insight into complex quantitative traits from the perspective of epigenetics.

Modern breeding techniques rely on mutation information at a genomic level. Differences in epigenetic status have a direct effect on expression and even phenotypes. It is difficult to detect the results of high-dimensional interactions between genes at the high-density chips and resequencing levels. Thus large-scale epigenetics data facilitates the analysis of complex quantitative traits and helps to achieve epigenetic breeding. Our data showed differential methylation and expression in LD between LW and BMX. The results supported that HOXC gene cluster plays an important role in maintaining and regulating specific muscle functions between breeds. *HOXC* cluster displayed myogenic hypermethylation bordering a central region containing many genes preferentially expressed in myogenic progenitor cells [43]. A subset of muscles may require HOXC8 protein for full activation of muscle-specific gene expression [29]. *HOXC8*, *ZIC1*, and *BMP5* are highly expressed in LD of LW pigs. The genes’ functions are not beneficial for accumulating IMF, but increasing trunk muscle mass which corresponds with lower IMF content and larger eye muscle area in LW pigs. Epigenetic differences between breeds are important factors that contribute to breed differentiation, synergistic effect or opposite causality. There is a stable difference in methylation levels between breeds, and clearly there is a stable genetic mechanism to maintain this feature. Clear mechanisms will bring about a qualitative change in breeding field. In addition, the differences between breeds imply that part of methylation characteristics which are present in tissue can be stably inherited within breed.

Although population genetics indicates that DNA methylation levels have moderate heritability [27], rare studies indicate that methylation levels are inherited or reproduced in future generations [25, 26]. The mammalian genome undergoes two extensive waves of reprogramming of CpG methylation during embryogenesis [1], but a fraction of mammalian genomes might potentially bypass the removal of DNA methylation [25]. Whereas de novo DNA methylation can occur in any sequence context, only symmetrical CpG methylation is maintained upon DNA replication [1]. We identified dozens of DMRs between breeds or pedigrees, which provided direct evidences that the methylation pattern is passed on to or reproduced in future generations. We propose two different hypotheses for tissue-specific DMRs between pedigrees. First, partial paternal genomes bypass the removal of DNA methylation, and it will be preserved in special differentiated tissues. Second, recurring DNA methylation in offspring does not depend on parental gamete methylation, it may be mediated by other unknown genetic mechanisms. The mechanism of intergenerational or transgenerational heritability needs to be further studied. We speculate the similar methylation pattern across generations are left markers from the progenitor and maybe play some biological roles.

## CONCLUSIONS AND PERSPECTIVE

The systematic exploration of dynamic methylomic landscapes has laid an important theoretical foundation for medical models. In this study, we drew dynamic methylome atlas using WGBS for different breeds, tissues, ages and genders. There are negative or positive correlation between methylation in DMRs and gene expression which are involved in tissue specific functions. DNA methylation modification plays an important regulatory role in different tissues and breeds and even brings about qualitative changes in phenotypes (*HOXC8* and *HOXC11*). The results of this study not only comprehensively investigated pig genome wide dynamic CpGs, but also established links between epigenetic, transcriptional, and phenotypic data. Importantly, personalized methylated or demethylated features can be passed on to future generations. The information will be useful for pig breeding and human disease. Rapid update of single molecule and single cell sequencing technologies make it possible to combine chromatin accessibility, methylome, and transcriptome in the same cell. It provides essential means for a further insight into the effect of DNA methylation on expression and phenotypes.

## MATERIALS AND METHODS

### Animals and sampling

All procedures involving animals are in compliance with guidelines for the care and use of experimental animals established by the Ministry of Agriculture of China, and the trial was approved by the Animal ethics committee at Jiangxi Agricultural University (No. JXAULL-2016001).

This study involved two pig breeds with dramatic genetic differences, one is western commercial purebred of Large White (LW) and the other is Chinese indigenous purebred of Bamaxing (BMX). Four LW pigs (about seven months, two virgin female and two male pigs) and eight male BMX pigs (including three nine-year-old (9Y), three four-year-old (4Y), and two one-year-old (1Y) pigs). They were raised at the farm and were fed on a similar diet, and they could access water and food *ad libitum* under a standardized feeding and management regimen. The pedigree information of BMX is showed in Supplementary Figure 1. After slaughting within postmortem 45 min, about 1 gram (g) portion of *Longissimus dorsi* (LD) muscle at the 1^st^-2^nd^ lumbar vertebra on the left side of the carcass and about 1g testis at central position of left testis were sampled and rapidly stored in −80° C refrigerator for DNA extraction.

### Whole genome bisulfite sequencing

A total of 2.26 Tb (average 113.20 Gb) WGBS data were generated using Hiseq X10 platform for twelve skeletal muscles and eight testes. The average mapping coverage and depth rate were 2.43 Gb and 46.49X per sample, respectively. The clean reads were aligned to the *Sus scrofa* 11.1 using bwa-meth-master, and an average of 96.92% reads were uniquely aligned to the reference genome. MethylDackel0.3.0 was used to summary methylation state and a total of 55,685,213 autosomal CpGs have been identified in at least one of twenty samples. These CpGs were annotated using Sus_scrofa.Sscrofa11.1.98.gtf which was downloaded from Ensembl (http://asia.ensembl.org/index.html).

### RNAseq

An average of 10G RNA-seq clean reads using NovaSeq5000 platform from 20 samples were mapped to *Sus scrofa* 11.1 with hisat2.1.0. The mapped reads were further analyzed by StringTie1.3.6 and subread1.6.5, and the expression levels for each transcript were quantified as fragments per kilobase of transcript per million mapped reads (FPKM).

### Differential analysis

CpGs with the coverage of less than 10 and greater than 300, and CpGs with individual missing rate less than 50% were filtered out. Three methods including MACAU [44], MALAX [45], and PQLseq [46] have been introduced for differential analysis. These methods adopted a binomial generalized linear mixed model (BGLMM) for count-based bisulfite sequencing data, but they used different algorithms including markov chain mote carlo (MCMC) sampling-based strategy [44], Laplace approximation [45], and penalized quasi-likelihood approach [46], respectively. The model treats age as a covariate and considers the covariance among individuals due to individual relatedness (Supplementary Table 5). The significant threshold was set as 0.01, the intersection result of three methods was used as DMCpGs. DMR was defined as combined sites within 500 bp of one another [15].

We also adopted a previously published method [14] which is based on the beta distribution to model single CpG methylation levels and exhibited a minimum methylation level difference of ≥0.3 at a significant level of *P*≤0.01. We estimated the posterior distribution of methylation levels in both genders from LW and three age groups from BMX [14], and we tested the minimum methylation level differences.

### Functional annotation

Pig methylation sites were annotated using Sus_scrofa.Sscrofa11.1.98.gtf, and the annotation of CGIs of *Sus scrofa* 11.1 reference sequence was downloaded from the University of California Santa Cruz (UCSC) Genome Browser. It was annotated following a criterion of segment length >200 bp, CG content >50%, and observed/expected ratio of CpG sites >0.6 [47]. CGI-shores are defined as the ~2 kb regions near CGI, and CpG shelves are further 2kb extension regions of CGI-shore. Differentially methylated gene lists were submitted to online tool DAVID (https://david.ncifcrf.gov/). We utilized *homo sapiens* set as “background” and selected GO terms and KEGG terms based on the statistical significant level (*P*<0.05).

### Inheritable methylation regions

The heritable region was defined as a 500 bp DMR in which at least 5 synclastic DMCpGs were detected for all individuals between pedigrees. In statistics, it is a very strict threshold (*P_fp_* value after Bonferroni correction is 0.01) to exclude random false positive. *P_fp_* value was calculated as follows:

The number of DMCpGs (N) within segments (L bp) follows a binomial distribution Bin(N, ECG, p/2).

$$P(X=N)=C_{ECG}^{N}{\left(\frac{p}{2}\right)}^{N}{\left(1-\frac{p}{2}\right)}^{ECG-N}$$

The expected number of CpGs (ECG):

$$ECG=\frac{Number\;of\;C\times Number\;of\;G}{L}$$

The false positive probability by Bonferroni correction:

$$P_{fp}(X\geq N)=2(1-P(X<N))\frac{GS}{L}$$

The ratio of DMCpG (*p*) is less than 0.003 between pedigrees in our data; The expected number of CpGs (ECG) is about 31 in a 500bp (L) random sequence; Pig genome size (GS) is about 2.5G. Under the ECG, the Bonferroni correction P value is:

$$P_{fp}(X\geq 5)=0.012$$

## Supplementary Material

Supplementary Figures

Supplementary Table 1

Supplementary Table 2

Supplementary Table 3

Supplementary Tables 4 and 5
