# Supplementary material for: DNA methylomic homogeneity and heterogeneity in muscles and testes throughout pig adulthood
Source: Aging (Albany NY). 2020 Nov 20;12(24):25412–31. doi: 10.18632/aging.104143 (PMC7803572; doi:10.18632/aging.104143)
Supplement: Supplementary Figures [file aging-12-104143-s001.pdf]

Supplementary Figures

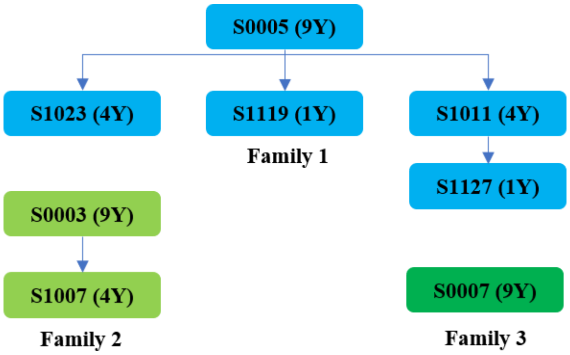

Supplementary Figure 1. The relationship and ages of BMX samples.

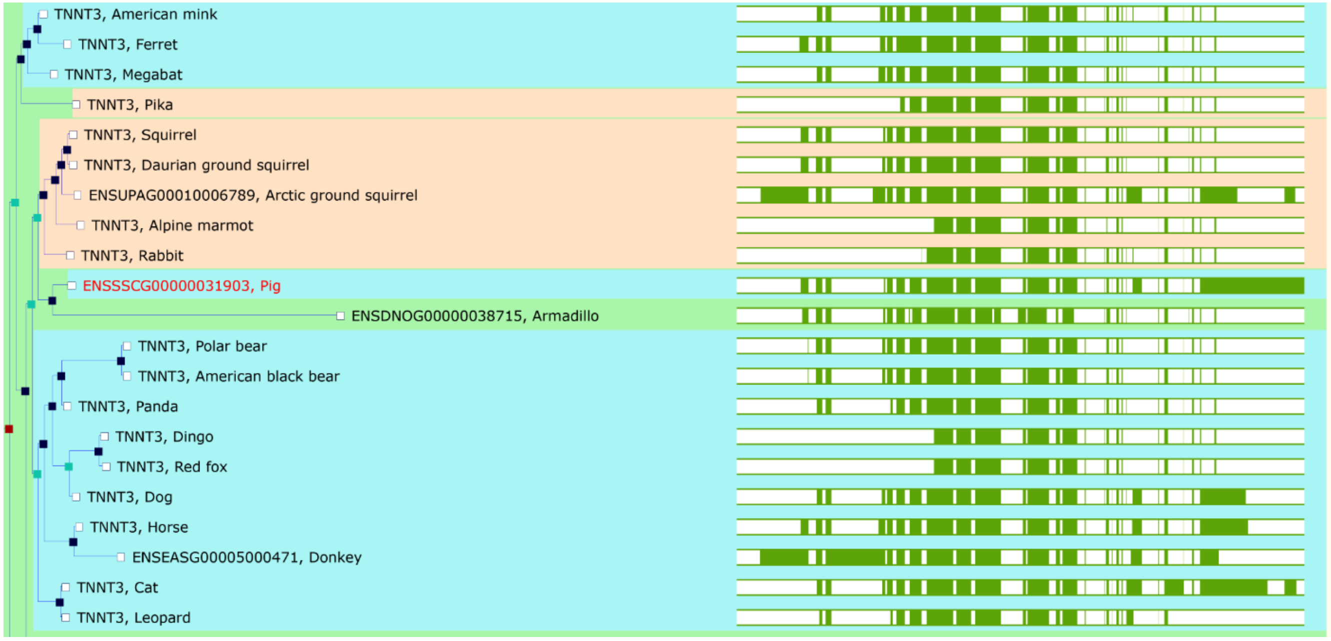

Supplementary Figure 2. The phylogenetic analyses of *ENSSSCG00000031903*.

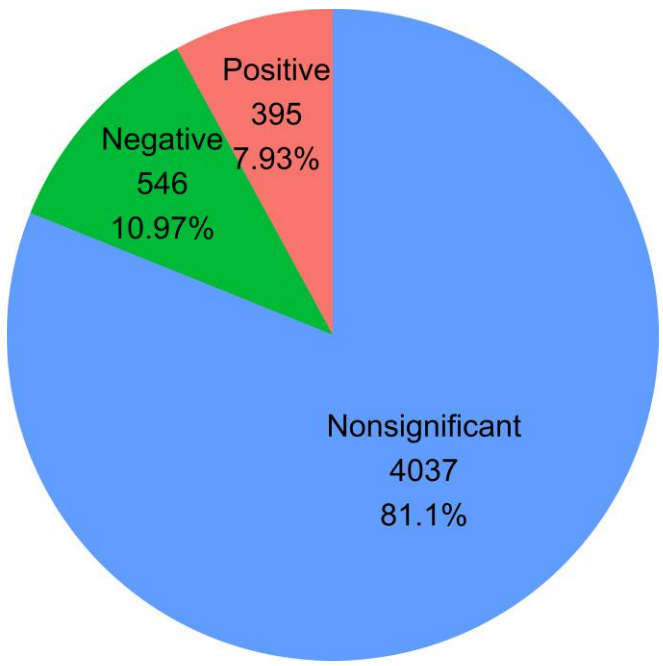

Supplementary Figure 3. The percent of genes whose expression levels are significantly correlated with DMCpGs.

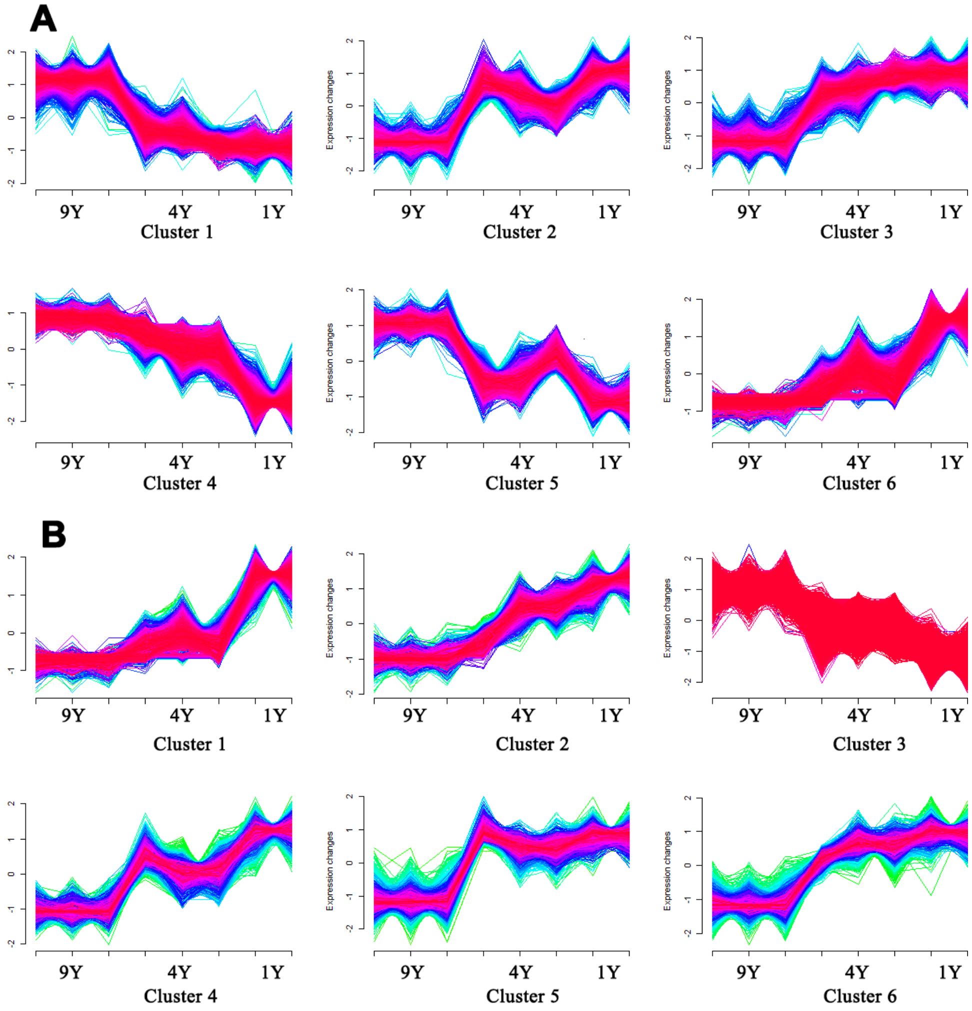

Supplementary Figure 4. Dynamic methylation patterns with age in muscles and testes. (**A**) Dynamic CpG patterns in muscles with age. (**B**) Dynamic CpG patterns in testes with age.
