# Supplementary material for: DNA methylomic homogeneity and heterogeneity in muscles and testes throughout pig adulthood
Source: Aging (Albany NY). 2020 Nov 20;12(24):25412–31. doi: 10.18632/aging.104143 (PMC7803572; doi:10.18632/aging.104143)
Supplement: Supplementary Table 1 [file aging-12-104143-s002.pdf]

Supplementary Table

Supplementary Table 1. The distribution of CpGs over ten regions.

Term	All	3' UTR	5' UTR	5'TUR - CGI	CGI	CGIshore	CGIshelves	Off CGI	Exon	Intron	Intergenic
All autosomal	55685213	1144772	939050	572002	5357203	6480234	2973362	40874414	5074619	28903302	21707292
Raw ratio	1	0.0206	0.0169	0.0103	0.0962	0.1164	0.0534	0.7340	0.0911	0.5190	0.3898
Filtered CpGs	34797932	655691	287284	62051	776576	3250115	1753194	29018047	2160187	18411778	14225967
Filtered ratio	1	0.0188	0.0083	0.0018	0.0223	0.0934	0.0504	0.8339	0.0621	0.5291	0.4088
lower CpG	1147532	16818	88349	55218	326513	341143	26826	453050	170735	516505	460292
Ratio	0.0330	0.0147	0.0770	0.0481	0.2845	0.2973	0.0234	0.3948	0.1488	0.4501	0.4011
higher CpG	5743199	145592	32157	1187	92375	485406	329421	4835997	464017	3754422	1524760
Ratio	0.1650	0.0254	0.0056	0.0002	0.0161	0.0845	0.0574	0.8420	0.0808	0.6537	0.2655
tissues	4283350	78915	29870	1575	82321	472590	217902	3510537	269853	2206777	1806720
Ratio	0.1231	0.1204	0.1040	0.0254	0.1060	0.1454	0.1243	0.1210	0.1249	0.1199	0.1270
breeds	1147591	20396	7053	746	41244	121052	67001	918294	69177	635685	442729
Ratio	0.0330	0.0311	0.0246	0.0120	0.0531	0.0372	0.0382	0.0316	0.0320	0.0345	0.0311
