# Supplementary material for: DNA methylomic homogeneity and heterogeneity in muscles and testes throughout pig adulthood
Source: Aging (Albany NY). 2020 Nov 20;12(24):25412–31. doi: 10.18632/aging.104143 (PMC7803572; doi:10.18632/aging.104143)
Supplement: Supplementary Tables 4 and 5 [file aging-12-104143-s005.pdf]

## SUPPLEMENTARY TABLES

**Supplementary Table 4. DMRs between pedigrees in muscle and testis.**

| Order | Tissue | Number | chr | Start | End |
|---|---|---|---|---|---|
| 1 | muscle | 7 | chr14 | 132993657 | 132994115 |
| 2 | muscle | 6 | chr8 | 137555650 | 137556000 |
| 3 | muscle | 6 | chr14 | 139325615 | 139325622 |
| 4 | muscle | 6 | chr14 | 134435262 | 134435655 |
| 5 | muscle | 5 | chr9 | 133567706 | 133568064 |
| 6 | muscle | 5 | chr6 | 91043485 | 91043970 |
| 7 | muscle | 5 | chr2 | 9089167 | 9089395 |
| 8 | muscle | 5 | chr2 | 47019679 | 47019922 |
| 9 | muscle | 5 | chr17 | 3180528 | 3180816 |
| 10 | muscle | 5 | chr14 | 139177374 | 139177532 |
| 11 | muscle | 5 | chr14 | 135865240 | 135865613 |
| 12 | muscle | 5 | chr12 | 19603524 | 19604001 |
| 13 | muscle | 5 | chr12 | 13733513 | 13733579 |
| 14 | muscle | 5 | chr11 | 8690188 | 8690624 |
| 15 | muscle | 5 | chr11 | 8539607 | 8539965 |
| 16 | muscle | 5 | chr11 | 11704769 | 11704816 |
| 17 | muscle | 5 | chr10 | 51583348 | 51583562 |
| 18 | testis | 9 | chr9 | 212771 | 212893 |
| 19 | tests | 9 | chr2 | 151574805 | 151575091 |
| 20 | testis | 7 | chr4 | 106091605 | 106091699 |
| 21 | testis | 7 | chr14 | 28641275 | 28641374 |
| 22 | testis | 7 | chr11 | 77646658 | 77646933 |
| 23 | testis | 7 | chr1 | 272100179 | 272100279 |
| 24 | testis | 6 | chr6 | 28480020 | 28480147 |
| 25 | testis | 6 | chr3 | 36920813 | 36921180 |
| 26 | testis | 6 | chr15 | 56009163 | 56009234 |
| 27 | testis | 6 | chr14 | 134435262 | 134435655 |
| 28 | testis | 6 | chr14 | 133842967 | 133843194 |
| 29 | testis | 6 | chr13 | 199879841 | 199880019 |
| 30 | testis | 6 | chr13 | 145939217 | 145939337 |
| 31 | testis | 6 | chr12 | 37030932 | 37031353 |
| 32 | testis | 6 | chr11 | 51716401 | 51716819 |
| 33 | testis | 5 | chr9 | 43645730 | 43646210 |
| 34 | testis | 5 | chr9 | 133631712 | 133632099 |
| 35 | testis | 5 | chr9 | 130114358 | 130114527 |
| 36 | testis | 5 | chr8 | 25277540 | 25277627 |
| 37 | testis | 5 | chr7 | 94740385 | 94740732 |
| 38 | testis | 5 | chr7 | 4854047 | 4854157 |
| 39 | testis | 5 | chr7 | 1120310 | 1120435 |
| 40 | testis | 5 | chr6 | 81718383 | 81718863 |
| 41 | testis | 5 | chr6 | 461361 | 461433 |
| 42 | testis | 5 | chr6 | 43618518 | 43618845 |
| 43 | testis | 5 | chr6 | 152254780 | 152255169 |
| 44 | testis | 5 | chr5 | 93255108 | 93255188 |
| 45 | testis | 5 | chr5 | 9054371 | 9054615 |
| 46 | testis | 5 | chr5 | 83501529 | 83501851 |

| | | | | | |
|---|---|---|---|---|---|
| 47 | testis | 5 | chr4 | 62328215 | 62328295 |
| 48 | testis | 5 | chr4 | 41868339 | 41868461 |
| 49 | testis | 5 | chr4 | 129351476 | 129351937 |
| 50 | testis | 5 | chr3 | 1702150 | 1702289 |
| 51 | testis | 5 | chr3 | 16255470 | 16255535 |
| 52 | testis | 5 | chr3 | 110508569 | 110508776 |
| 53 | testis | 5 | chr2 | 88990833 | 88990886 |
| 54 | testis | 5 | chr2 | 6815401 | 6815852 |
| 55 | testis | 5 | chr2 | 59295363 | 59295652 |
| 56 | testis | 5 | chr2 | 39226566 | 39226744 |
| 57 | testis | 5 | chr2 | 1658708 | 1658848 |
| 58 | testis | 5 | chr2 | 140548352 | 140548438 |
| 59 | testis | 5 | chr18 | 2505488 | 2505932 |
| 60 | testis | 5 | chr17 | 9761541 | 9761933 |
| 61 | testis | 5 | chr17 | 29865514 | 29865914 |
| 62 | testis | 5 | chr16 | 49558122 | 49558191 |
| 63 | testis | 5 | chr16 | 45044933 | 45045071 |
| 64 | testis | 5 | chr16 | 3671532 | 3671693 |
| 65 | testis | 5 | chr14 | 9907095 | 9907265 |
| 66 | testis | 5 | chr14 | 73624262 | 73624403 |
| 67 | testis | 5 | chr14 | 138098493 | 138098957 |
| 68 | testis | 5 | chr14 | 136637468 | 136637968 |
| 69 | testis | 5 | chr13 | 73621572 | 73621803 |
| 70 | testis | 5 | chr13 | 207854405 | 207854841 |
| 71 | testis | 5 | chr12 | 6090884 | 6091214 |
| 72 | testis | 5 | chr12 | 4379572 | 4379698 |
| 73 | testis | 5 | chr12 | 18924477 | 18924618 |
| 74 | testis | 5 | chr12 | 13967609 | 13967737 |
| 75 | testis | 5 | chr11 | 723913 | 723951 |
| 76 | testis | 5 | chr11 | 5193167 | 5193383 |
| 77 | testis | 5 | chr11 | 4588027 | 4588100 |
| 78 | testis | 5 | chr11 | 11668788 | 11668941 |
| 79 | testis | 5 | chr10 | 51629956 | 51630323 |
| 80 | testis | 5 | chr10 | 20867433 | 20867532 |
| 81 | testis | 5 | chr1 | 207728 | 207779 |

**Supplementary Table 5. Sample size, covariates and covariances in BGLMM.**

| Analysis | Sample size | Covariate | Covariance |
|---|---|---|---|
| Ages (muscle) | 8 (2 1Ys, 3 4Ys, and 3 9Ys) | | relatedness |
| Ages (testis) | 8 (2 1Ys, 3 4Ys, and 3 9Ys) | | relatedness |
| Tissues | 8 (muscle) vs 8 (testis) | ages | relatedness |
| Breeds | 4 (LW) vs 8 (BMX) | | |
| Pedigrees | 2 (BMX) vs 5 (BMX) | | |
| Genders | 2 (male LW) vs 2 (female LW) | | |
